# Chronic loneliness and isolation phenotypes, incident functional impairment and mortality in England between 2004 and 2018

**DOI:** 10.1038/s44220-025-00436-0

**Published:** 2025-05-19

**Authors:** Qian Gao, Andrew Steptoe, Daisy Fancourt

**Affiliations:** 1 School of Public Health, Imperial College London, London, UK; 2 Department of Behavioural Science and Health, Institute of Epidemiology and Health Care, University College London, London, UK

**Keywords:** Epidemiology, Public health

## Abstract

Social deficits are potential risk factors for premature mortality. Most research has focused on social deficits measured at single points in time. It remains unclear if the chronicity of loneliness affects its impact on adverse health outcomes. This study assessed the effects of chronic loneliness and isolation in predicting incident functional impairment and all-cause and cause-specific mortality. This longitudinal study used panel data from the English Longitudinal Study of Ageing, including 14 years of follow-up (waves 2–9, in 2004–2018). Social deficits over three waves (4 years) were measured using the UCLA loneliness scale and social isolation index, categorized as not present, fluctuating or chronic. We estimated the all-cause mortality risk with Cox proportional hazard modeling, and the Fine–Gray competing risk modeling was used to test the risk of functional impairment onset and cause-specific mortality. We analyzed 5,131 participants (mean age 67.6 years (s.d. 9.8)) in the mortality cohort (follow-up 9.8 years (IQR: 6.67–10.08)) and 4,279 participants (mean age 67.0 years (s.d. 9.6)) who were functional disability-free at baseline (follow-up 9.8 years (IQR: 7.17–10.17)). Compared with not being lonely/isolated, there was a higher risk of incident functional impairment among those with fluctuating loneliness (sub-hazard ratio (sHR) 1.30, 95% CI: 1.03–1.63) and chronic loneliness (sHR 1.58, 1.12–2.23), as well as chronic social isolation (sHR 1.41, 1.02–1.94). In survival analyses, compared with people who were not lonely/isolated, people experiencing fluctuating loneliness and social isolation had higher risks of all-cause mortality (loneliness HR 1.29, 1.13–1.48; isolation HR 1.15, 1.01–1.31). People with chronic isolation also had higher risks of all-cause mortality (HR 1.27, 1.05–1.55) and cancer-related mortality (sHR 1.69, 1.23–2.31). Over a 14 year follow-up, we found that chronic loneliness and isolation phenotypes were associated with aggravated risks of incident functional impairment and mortality. There was a potential dose–response relationship between chronicity of loneliness phenotypes and functional impairment onset and mortality. Preventing the onset of and transition to chronic loneliness and isolation in older age is a crucial target to support both the healthspan and the lifespan.

## Abstract (web summary)

Chronic loneliness and isolation significantly increase risks of functional impairment and mortality, as shown through longitudinal analysis of 5,131 participants using Cox proportional hazards and Fine–Gray models, highlighting the importance of addressing these social deficits in older adults.

## Main

Deficits in social relationships have physical and mental health consequences that pose challenges to public health systems. This problem is becoming greater for societies as some aspects of social relationships (such as isolation) can increase as people age, and in some cultures, social engagement has been decreasing over the past two decades^1^. It is estimated that 18–29% of people over the age of 60 live with loneliness or social isolation in the United Kingdom^2,3^. Loneliness is conceptualized as a subjective dissatisfaction with social connectedness and relationships^4^, while social isolation objectively measures the absence of proper social interaction and connection^5^. Both subjective loneliness and objective isolation have been recognized as key components of the biopsychosocial frailty phenotype, which adversely affect the development and progression of chronic diseases, such as cardiovascular diseases, late-life cognitive impairment and dementia^6,7^, as well as impacting the incidence and progression of mental illnesses such as anxiety and depression and affecting functional capabilities^5,8^. All of these conditions have consequences for health systems at all levels, increasing health-care consumption of primary care^9^, outpatient care^10,11^ and rehospitalization^10^.

To date, much of the research in this space has focused on social deficits without considering the length of time that individuals have experienced them. But chronic loneliness and isolation phenotypes may play a more robust role in shaping adverse health consequences, which could differ from acute and occasional loneliness^12^. Indeed, the evolutionary theory of loneliness posits that lonely or socially isolated individuals are likely to hold negative expectations of meeting social needs and enhanced social deficits, resulting in cycles of more intense and persistent loneliness and isolation^13^. Long-term exposure to loneliness and/or social isolation is linked to biosocial pathogenesis^14,15^, and the health hazards can accumulate through persistent psychological stress and unhealthy behaviors^16^. For example, as a persistent psychological stressor, chronic loneliness may result in allostatic overload^17–19^ and long-term stress system overactivation^20^, as well as dysregulated inflammatory processes^21,22^ and upregulated gene transcription of pro-inflammatory control pathways^23^.

There is growing research suggesting that these biological pathways involved in chronic social deficits could have implications for the healthspan and lifespan. Several studies have suggested that chronic loneliness is a more harmful predictor of cardiovascular health (for example, lower resting heart rate variability)^24^ and incident coronary disease^25^ than acute loneliness. Older adults experiencing social deficits are also vulnerable to earlier and faster functional declines^8,26^ (setting in motion feedback loops that in turn detrimentally affect social interactions^27^). Social connections appear to help older people remain independent^28^. However, it remains unclear whether chronic loneliness and/or social isolation can drive a higher incidence of functional impairment. Further, previous epidemiological research from multiple countries has identified an increased all-cause mortality risk among older people who report subjective loneliness^26,29–31^ or objective social isolation^32,33^. Again, however, it is unclear whether this risk is higher among older adults with chronic social deficits.

Therefore, this study aimed to address this research gap by examining the roles of chronic loneliness and social isolation in predicting the onset of physical functional declines and all-cause and cause-specific mortality over 10-year follow-ups using a nationally representative sample of older adults living in England.

## Results

### Participants

The study consisted of 4,279 participants in the functional disability-free cohort (mean age 67.0 years, s.d. 9.6) and 5,131 participants in the mortality cohort (mean age 67.6 years, s.d. 9.8; Table 1). The disability-free samples had a 20.2% all-cause mortality rate, with 9.83 years of median follow-up (interquartile range (IQR): 7.17–10.17). The all-cause mortality rate was 22.4% in the mortality cohort, with a median follow-up length of 9.83 years (IQR: 6.67–10.08).

**Table 1:** Characteristics of study samples

Characteristics	Functional disability-free cohort (*n* = 4,279)	Mortality cohort (*n* = 5,131)
**Age (mean (s.d.))**	67.0 (9.6)	67.6 (9.8)
**Sex**		
Female	2,379 (55.6%)	2,868 (55.9%)
Male	1,900 (44.4%)	2,263 (44.1%)
**Education**		
No or basic qualifications	1,337 (31.3%)	1,735 (33.8%)
General Certificate of Secondary Education/O level/qualification at age 16	1,172 (27.4%)	1,356 (26.4%)
A levels/higher education/qualification at age 18	1,010 (23.6%)	1,195 (23.3%)
Degree/further higher qualification	760 (17.7%)	845 (16.5%)
**Household income (mean (s.d.))**	339.3 (262.9)	330.1 (249.3)
**Employment**		
Unemployed or retired	2,862 (66.9%)	3,626 (70.7%)
Actively working	1,417 (33.1%)	1,505 (29.3%)
**Sedentary lifestyle**		
Less than weekly	620 (14.5%)	1,039 (20.3%)
Weekly and more frequent	3,659 (85.5%)	4,092 (79.7%)
**Chronic illness**		
Yes	2,110 (49.3%)	2,877 (56.1%)
None	2,169 (50.7%)	2,254 (43.9%)
**Persistent pain**		
Yes	926 (21.6%)	1,467 (28.6%)
No	3,353 (78.4%)	3,664 (71.4%)
**Depression**		
Yes	819 (19.1%)	1,177 (22.9%)
No	3,460 (80.9%)	3,954 (77.1%)
**Loneliness**		
No loneliness	2,617 (61.2%)	3,028 (59.0%)
Fluctuating loneliness	1,427 (33.3%)	1,767 (34.4%)
Chronic loneliness	235 (5.5%)	336 (6.6%)
**Social isolation**		
No isolation	2,257 (52.8%)	2,682 (52.3%)
Fluctuating isolation	1,662 (38.8%)	1,970 (38.4%)
Chronic isolation	360 (8.4%)	479 (9.3%)
**Baseline functional impairment**		
ADLs disability	0	1,019 (19.9%)
No disability	4,279 (100%)	4,112 (80.1%)
**Median length of follow-up** **(years (IQR))**	9.83 (7.17–10.17)	9.83 (6.67–10.08)
**Mortality rate per 1,000 person-years**	24.22 (22.65–25.89)	27.14 (25.61–28.76)
**All-cause death**	864 (20.2%)	1,148 (22.4%)
**Cause-specific death**		
Cancer	314 (7.3%)	385 (7.5%)
CVD	245 (5.7%)	333 (6.5%)
Respiratory disease	126 (3.0%)	174 (3.4%)
Other cause	179 (4.2%)	256 (5.0%)

ADL, activity of daily living.

In the mortality cohort, the prevalence of chronic loneliness and isolation were 6.6% and 9.3%, respectively; 34.4% of the participants had fluctuating loneliness, and 38.4% had fluctuating isolation. Chronic loneliness and isolation were slightly less prevalent in the disability-free cohort, with 5.5% and 8.4% of the sample reporting the issues.

### Incident functional impairment

Compared with no loneliness, chronic loneliness (sub-hazard ratio (sHR): 1.58, 95% confidence interval (CI) 1.12–2.23) and fluctuating loneliness (sHR: 1.30, 1.03–1.63) were associated with increased risk of incident functional impairment (Table 2). These results were found when adjusting for social confounders and were maintained (albeit slightly attenuated) when additionally adjusting for health-related confounders. Fluctuating social isolation was not consistently associated with functional impairment when adjusting for confounders, but older adults with chronic social isolation had higher risks of developing new functional impairment cases (sHR: 1.41, 1.02–1.94).

**Table 2:** Associations between chronic and fluctuating loneliness and social isolation and incident functional impairment

Incident functional impairment	Loneliness (sHR, 95% CI)		Social isolation (sHR, 95% CI)	
	Fluctuating loneliness	Chronic loneliness	Fluctuating social isolation	Chronic social isolation
Unadjusted	1.77 (1.44–2.17)	2.39 (1.73–3.32)	1.53 (1.24–1.88)	1.88 (1.37–2.59)
Model 1	1.61 (1.30–1.98)	2.06 (1.47–2.88)	1.22 (0.98–1.52)	1.57 (1.14–2.17)
Model 2	1.30 (1.03–1.63)	1.58 (1.12–2.23)	1.09 (0.88–1.36)	1.41 (1.02–1.94)

Reference group: no loneliness/social isolation. We conducted the Fine–Gray competing risk model. Model 1 was adjusted for age, sex, education, household income, employment and sedentary lifestyle; Model 2 was additionally adjusted for depression, persistent pain and long-term illness.

### All-cause and cause-specific mortality

**Fig. 1:**
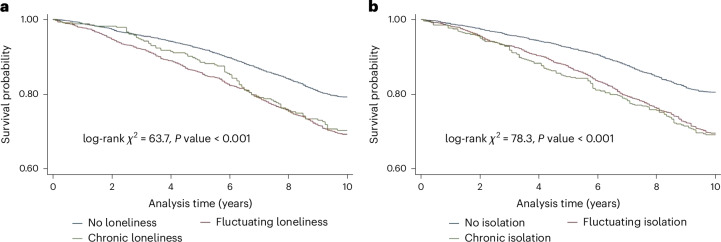
Kaplan–Meier survival curve for 10-year all-cause mortality by different patterns of loneliness and social isolation. **a**, Loneliness. **b**, Social isolation. Kaplan–Meier survival curves were graphed, and two-sided log-rank tests were conducted to compare the differences in curves across patterns of loneliness/isolation over 4 years.

In survival analyses, crude Kaplan–Meier curves showed that the fluctuating and chronic social isolation group had lower 10-year survival rates than the not isolated group (Fig. 1), with evidence of a slight dose–response relationship depending on chronicity. The pattern for loneliness was less clear, with a clear higher survival rate for the not lonely group but inconsistent patterns over time between fluctuating and chronic loneliness and survival rates. The results were consistent in cause-specific mortality analyses (Supplementary Fig. 1).

In the fully adjusted Cox proportional model, compared with no social isolation, chronic isolation had the most elevated all-cause mortality risks (hazard ratio (HR) 1.27, 1.05–1.55), followed by fluctuating isolation (HR 1.15, 1.01–1.31) (Table 3). Fluctuating loneliness had a higher all-cause mortality risk than no loneliness (HR 1.29, 1.13–1.48). However, the adverse effects of chronic loneliness on all-cause mortality were attenuated after adjustment for health-related confounders. In the cause-specific mortality analyses, social isolation (especially chronic social isolation) was related to higher cancer-related mortality risk and respiratory-related mortality risk, while loneliness was related to higher cardiovascular disease (CVD) mortality (although results were less robust for chronic loneliness).

**Table 3:** Associations between changes in loneliness and social isolation, and all-cause and cause-specific mortality

All-cause mortality	Loneliness		Social isolation	
	Fluctuating loneliness, HR	Chronic loneliness, HR	Fluctuating social isolation, HR	Chronic social isolation, HR
Unadjusted	1.61 (1.43–1.82)	1.51 (1.21–1.89)	1.68 (1.48–1.90)	1.74 (1.44–2.11)
Model 1	1.46 (1.29–1.66)	1.32 (1.05–1.65)	1.22 (1.07–1.38)	1.38 (1.14–1.67)
Model 2	1.29 (1.13–1.48)	1.11 (0.87–1.40)	1.15 (1.01–1.31)	1.27 (1.05–1.55)
**Cancer mortality**				
Unadjusted	0.93 (0.75–1.16)	1.11 (0.75–1.64)	1.36 (1.10–1.69)	1.84 (1.35–2.51)
Model 1	0.93 (0.74–1.17)	1.09 (0.74–1.62)	1.27 (1.01–1.58)	1.72 (1.26–2.35)
Model 2	0.85 (0.67–1.08)	0.95 (0.62–1.44)	1.23 (0.98–1.54)	1.69 (1.23–2.31)
**CVD mortality**				
Unadjusted	1.79 (1.43–2.25)	1.94 (1.32–2.85)	1.78 (1.42–2.24)	1.48 (1.01–2.17)
Model 1	1.44 (1.13–1.84)	1.59 (1.05–2.41)	1.26 (0.98–1.61)	1.14 (0.77–1.68)
Model 2	1.29 (1.00–1.67)	1.38 (0.90–2.12)	1.18 (0.92–1.52)	1.07 (0.72–1.59)
**Respiratory disease mortality**				
Unadjusted	1.93 (1.42–2.64)	1.70 (0.96–2.99)	1.70 (1.23–2.36)	2.24 (1.43–3.53)
Model 1	1.47 (1.05–2.05)	1.49 (0.85–2.62)	1.17 (0.83–1.65)	1.66 (1.03–2.67)
Model 2	1.28 (0.90–1.81)	1.24 (0.69–2.25)	1.11 (0.79–1.57)	1.55 (0.96–2.48)

Reference group: no loneliness/social isolation. We applied the Cox proportional hazards model to estimate the effects of loneliness and social isolation on all-cause mortality and the Fine–Gray competing risk model to assess their roles in cause-specific mortality. The proportional hazard assumption was assessed using the Schoenfeld residuals test. Model 1 was adjusted for age, sex, education, household income, employment and sedentary lifestyle; Model 2 was additionally adjusted for depression, persistent pain and long-term illness.

## Discussion

Over the 14-year follow-up of a nationally representative sample of older adults in England, we found that social isolation and loneliness were both related to incident functional impairment and mortality risk. Chronic isolation and loneliness had stronger associations with both outcomes than fluctuating isolation and loneliness, suggesting that temporal patterns and longevity of these experiences are important risk factors. We saw differences in patterns of cause-specific mortality, with cancer and respiratory disease mortality linked more to social isolation and CVD mortality to loneliness, suggesting different bio-behavioral pathways. Results were consistent when accounting for sociodemographic factors. Some results were attenuated when taking into account health-related factors, which could indicate some partial mediation of associations. It is notable, however, that many of the key results were still maintained (especially for chronic social deficits) even when adjusting for these factors, highlighting that results are not merely due to pre-existing associations between social deficits and mental and physical health symptoms.

Notably, we provide new evidence that chronic loneliness and isolation are related to higher functional impairment and mortality risks than fluctuating patterns. The chronic phenotype appears more detrimental to health, with a dose–response relationship between chronicity and outcomes, suggesting that adverse health effects of loneliness and isolation may accumulate over time and drive poorer health outcomes^12^. Indeed, prolonged loneliness could cumulatively damage functional ability and longevity through long-term unhealthy behaviors (for example, sedentary lifestyle)^16^, prolonged stress system overactivation^20^ and chronic inflammation^21,23^. The slightly higher mortality risk for fluctuating subjective loneliness above chronic loneliness may have resulted from the small number of participants who reported chronic loneliness in our study.

Further, we found a distinction between loneliness and social isolation in cause-specific mortality, with social isolation (especially the chronic phenotype) associated with higher cancer-related mortality risk and respiratory-related mortality risk, while loneliness was related to higher CVD-related mortality. The loneliness–CVD findings support previous studies showing increased risk of CVD due to loneliness-based activation of psychological pathways (depression, stress, anger and hostility), adverse immune responses (atypical neuroendocrine stress responses, increased inflammation and enhanced oxidative stress), cardiometabolic pathways (blood pressure, heart rate variability, increased vascular resistance and changes in gut microbiota) and potential epigenetic changes, in addition to behavioral factors such as physical inactivity, body mass index and substance use^34^. Indeed, we have previously found that loneliness as opposed to social isolation is associated with increased hospital admissions for CVD events as well as increased risk for incident CVD outcomes, including incident CVD events^35^ and mortality^36^. Similarly, the social isolation results support past findings suggesting that social isolation (but not loneliness) increases the risk of respiratory disease admissions, potentially due to risk factors such as smoking^35,37,38^.

The findings for cancer do reflect some past literature failing to find associations between loneliness and cancer-related mortality (after adjusting for confounders)^39^. However, previous findings of a relationship for social isolation with cancer-related mortality are mixed^36,40^. Nonetheless, our findings of a relationship that is dose-dependent on chronicity could be due to some shared mechanistic pathways. Social isolation can influence cancer progression and survival through activated tumorigenic markers^41^, higher vascular endothelial growth factor and pro-inflammatory biomarkers that contributed to tumor angiogenesis^42,43^. However, these pathways can also be activated via loneliness, and it is notable that loneliness was not linked to cancer survival, suggesting additional mechanistic pathways are also at play. Instead, socially isolated individuals may receive less social encouragement to seek medical advice about early symptoms of cancer, leading to late cancer detection and a higher risk of developing advanced cancer. Similarly, a supportive social network and connections could potentially improve cancer outcomes through facilitating cancer recovery and functional independence (including adherence to cancer treatments)^44^. As socioemotional selectivity theory illustrates, time perception may affect social network and preferred social partners. For example, cancer survivors may prioritize social connections such as family communications in the wake of diagnoses^45^. While our research adjusted for existing chronic illness, future studies could seek to disentangle whether existing health conditions such as cancer lead people to adjust their social behaviors, potentially leading to a reverse-causal finding.

A key contribution of this study is in providing evidence on chronic social deficits, where previous studies have often relied on single time-point measurements. Consistent with previous findings^26,29,32,33^, both subjective loneliness and objective isolation were associated with increased premature mortality risk in this study. However, aligning with previous UK Biobank findings showing that isolation but not loneliness was related to higher all-cause mortality after full adjustment^46^, after accounting for health-related confounders, we found that chronic loneliness is no longer an independent risk factor for all-cause mortality, suggesting that the harmful effect of chronic subjective loneliness on mortality may be attributable to mental and physical health. Loneliness is related to worsening health status^5^, which could mediate the association between chronic loneliness and premature mortality. However, our findings suggest that people with chronic loneliness and social isolation are more likely to develop functional impairment onset, illustrating that chronic phenotypes may have a significant role in accumulating harmful effects on functional abilities. This confirms and supplements previous evidence that loneliness and social isolation are linked with worsening functional declines in later life^8,26^. Lacking a socially supportive environment can potentially hinder access to resources and develop coping strategies, leading to incident functional ability declines among older adults^44^.

This investigation in England provides insights into associations of chronic loneliness and social isolation with the incidence of functional impairment and all-cause and cause-specific mortality using a nationally representative sample. English Longitudinal Study of Ageing (ELSA) data provide comprehensive information on social demographic and health-related confounders. Nevertheless, there are several limitations in current estimates. First, this is an observational study, so causality cannot be demonstrated. Although we considered an extensive set of confounders, it is possible for residual confounding to remain. For example, our population-based survey data limited the possibility of taking into account clinically validated mental health diagnoses, many of which are likely to co-exist with loneliness and isolation phenotypes. We considered depression with a validated and widely applied scale (above-threshold depression scores) in the modeling, but we cannot rule out the potential overestimation/underestimation of the current effect sizes. Second, most of our measures were collected through self-report, which may introduce response bias. In addition, findings of cause-specific mortality analyses (three types of causes) may be affected by statistical power, so they are presented as exploratory. However, the results are consistent with all-cause mortality analyses. Finally, the magnitude of risk aggravation could also be varied depending on the different measurements of loneliness applied (single versus multiple item measures)^30^. Future studies could explore the consistency of findings in other studies with different measurement approaches.

## Conclusion

The study sheds light on the effects of chronic loneliness and social isolation on predicting incident functional impairment and premature mortality, highlighting the importance of addressing chronic loneliness phenotypes to better support healthy ageing. Our findings indicate that both subjective loneliness and objective social isolation might increase the risks of functional impairment and mortality risk, with social isolation (especially the chronic phenotype) associated with higher all-cause and cancer-related mortality risks and loneliness related to higher risks for all-cause and CVD-related mortality independent of sociodemographic and health status. Preventing the onset and transition to chronic loneliness phenotypes in older age is vital to facilitating disease management and recovery. Chronic loneliness and social isolation could be targets for policy and public health practice in the future, especially in developing interventions to help older people maintain functional ability and healthy longevity.

## Methods

### Study sample

ELSA was designed to recruit a representative sample of adults aged 50 years and older living in private households in England. The ELSA survey was started in 2002 and then followed up every 2 years^47^. In this study, we used data from wave 2 (in 2004) as this is the first wave that provides loneliness and social isolation measures. We initially included participants who had completed the loneliness/social isolation information in waves 2–4 (*n* = 6,004).

To explore the relationship with functional decline, we limited the sample to participants free of physical disabilities at baseline (wave 4; definition follows) (*n* = 4,857). To enable analysis of competing risks of disability with death, we then excluded those who did not consent to linkage with the UK Office for National Statistics data (*n* = 244) and with missingness in covariates (*n* = 334), leading to a final sample (*n* = 4,279).

To explore the relationship with mortality, we linked the records with the UK Office for National Statistics to ascertain death dates and vital status and excluded loss to follow-up samples (*n* = 274). The study also excluded those with missingness in covariates (*n* = 599), providing a final analytical sample of 5,131. Details of the study sample selection process are shown in Supplementary Fig. 2.

### Measures

#### Outcomes

Incident functional impairment was defined as developing impairments in one or more ADLs at follow-up waves (2010–2018), consisting of getting in or out of bed, walking across a room, bathing or showering, using the toilet, dressing (including putting on shoes and socks) and eating, such as cutting food^48^.

All-cause and cause-specific mortality data were ascertained from the National Health Service central data registry, including deaths from cancer, CVD, respiratory disease and other causes using ICD-10 (International Classification of Diseases, 10th Revision)^49^ codes. Vital status was followed up until March 2018.

#### Exposures

Loneliness was measured by a validated three-item version of the UCLA (University of California at Los Angeles) loneliness scale^50,51^, measuring whether individuals felt (1) lack of companionship, (2) left out and (3) isolated from others, with a sum score ranging from 3 to 9 and a cut-off score of ≥6 to indicate loneliness^2^.

For social isolation, we used the five-item index of social isolation, which captures social contactlessness in domains: (1) unmarried/not cohabiting or living alone, (2) no engagement in any groups, clubs or other social organizations and less than monthly contact (via face-to-face, phone or written/e-mail contact) with children, family members and friends. It has been applied and validated in ELSA^2,51,52^. The overall scores range from 0 to 5, and those with a score of ≥3 were categorized as socially isolated^50^.

In this study, we measured changes in loneliness/social isolation over the first three waves (2004–2008) and generated new three-category loneliness and social isolation variables: no loneliness/isolation (not lonely/socially isolated in any waves), fluctuating loneliness/isolation (reported loneliness/isolation in one or two waves) and chronic loneliness/isolation (reported loneliness/isolation in all waves).

#### Covariates

We considered baseline (wave 2, in 2004) sociodemographic characteristics, including age, sex (male, female), educational attainment (no qualifications or basic qualifications, General Certificate of Secondary Education or O level or qualification at age 16, A levels or higher education or qualification at age 18, degree or further higher qualification), equivalized household income^53^ and employment status (employed versus unemployed or retired). We also adjusted for health-behavior confounders such as sedentary lifestyle (less than weekly engagement versus weekly and more frequently engagement in mild, moderate or vigorous sports or activities).

We further considered that some health-related factors could act as confounders, including long-standing chronic illness (one or more of cancer, chronic obstructive pulmonary disease, arthritis, stroke, diabetes, angina), depression (measured using the eight-item Center for Epidemiologic Studies Depression Scale with a score of ≥3 to indicate depression (yes/no)^54^) and persistent pain (moderate or severe chronic pain (yes/no)). However, it is also plausible that all of these lie on the causal pathway; hence, adjusting for them could result in partial mediation of findings. Therefore, we included them only in a final model to compare the effects of their inclusion on the findings^55^.

### Statistical analysis

To examine the relationship with incident physical functional disability, we identified the onset of ADL disability as a ‘competing outcome’ in the functional disability-free cohort and conducted the Fine–Gray competing risk model^56^ (additionally adjusting for competing risk of death). To examine the relationship between social deficits and mortality, we applied the Cox proportional hazards model to estimate the effects of chronic loneliness and social isolation on all-cause mortality and the Fine–Gray competing risk model to assess their roles in cause-specific mortality. The proportional hazard assumption was assessed using the Schoenfeld residuals test. Kaplan–Meier survival curves were graphed, and log-rank tests were conducted to compare the differences in curves across patterns of loneliness/isolation over 4 years. In modeling, we adjusted for baseline age, sex, education, household income, employment and sedentary lifestyle at baseline (wave 4) in Model 1. Model 2 then additionally adjusted for baseline depression, persistent pain and long-term illness, but this was treated as a comparison model given the potential risk of partial mediation of findings. All analyses were performed using STATA 17.0.

### Ethics and inclusion statement

This work analyzed the English Longitudinal Study of Ageing. The research is locally relevant, and all co-authors are from the United Kingdom. All co-authors have agreed on their roles and responsibilities ahead of research. This research complies with the Declaration of Helsinki. ELSA Wave 9 received ethical approval from the South Central-Berkshire Research Ethics Committee on 10 May 2018 (17/SC/0588). ELSA Wave 8 received ethical approval from the South Central-Berkshire Research Ethics Committee on 23 September 2015 (15/SC/0526). ELSA Wave 7 received ethical approval from the National Research Ethics Service Committee South Central-Berkshire on 28 November 2013 (13/SC/0532). ELSA Wave 6 received ethical approval from the National Research Ethics Service Committee South Central-Berkshire on 28 November 2012 (11/SC/0374). ELSA Wave 5 received ethical approval from the Berkshire Research Ethics Committee on 21 December 2009 (09/H0505/124). ELSA Wave 4 received ethical approval from the National Hospital for Neurology and Neurosurgery and Institute of Neurology Joint Research Ethics Committee on 12 October 2007 (07/H0716/48). ELSA Wave 3 received ethical approval from the London Multi-Centre Research Ethics Committee on 27 October 2005 (05/MRE02/63). ELSA Wave 2 received ethical approval from the London Multi-Centre Research Ethics Committee on 12 August 2004 (MREC/04/2/006). Informed consent was acquired from all participants.

### Reporting summary

Further information on research design is available in the Nature Portfolio Reporting Summary linked to this article.

## Supplementary information

Supplementary Information Supplementary Figs. 1 and 2.

Reporting Summary

## Data Availability

ELSA data are available through registration with the UK data service (https://beta.ukdataservice.ac.uk/datacatalogue/series/series?id=200011).

## Code availability

All data analyses used open-source STATA code (that is, Cox proportional hazards model and Fine–Gray competing risk model).
