# Supplementary material for: Chronic loneliness and isolation phenotypes, incident functional impairment and mortality in England between 2004 and 2018
Source: Nat Ment Health. 2025 May 19;3(6):667–74. doi: 10.1038/s44220-025-00436-0 (PMC12165843; doi:10.1038/s44220-025-00436-0)

nature mental health

Supplementary information

https://doi.org/10.1038/s44220-025-00436-0

Chronic loneliness and isolation phenotypes, incident functional impairment and mortality in England between 2004 and 2018

In the format provided by the authors and unedited

Supplementary Figure 1 | Kaplan-Meier survival curve for 10-year cause-specific mortality by different patterns of loneliness and social isolation

a

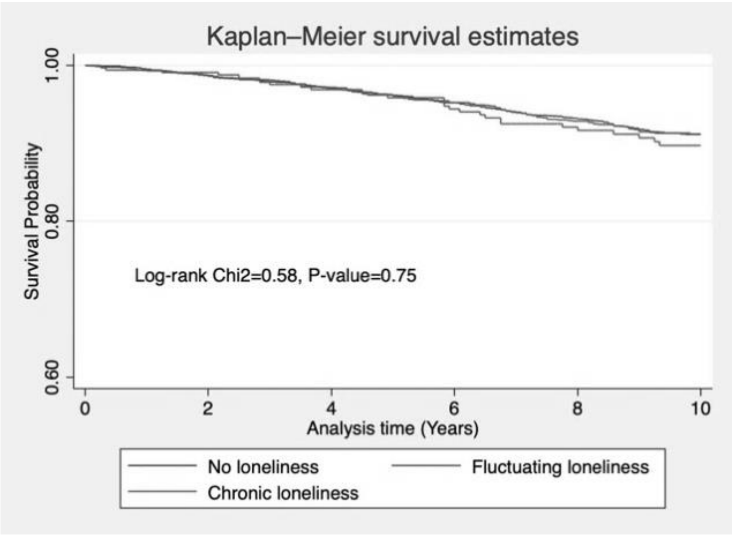

b

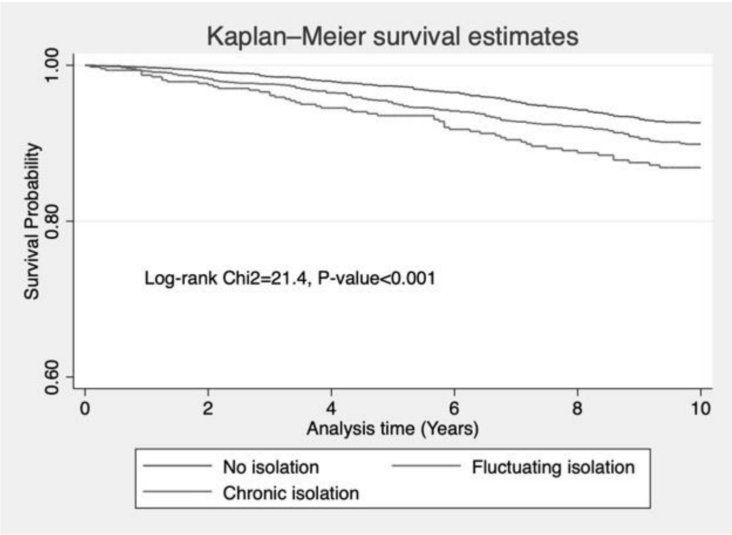

c

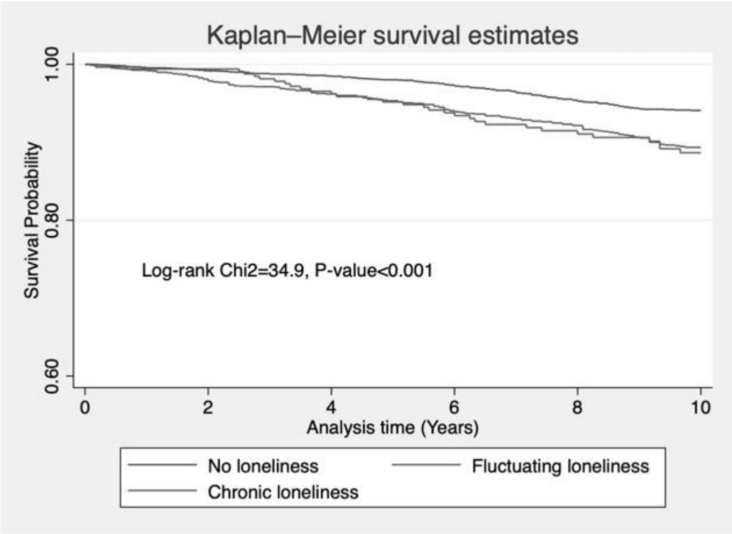

d

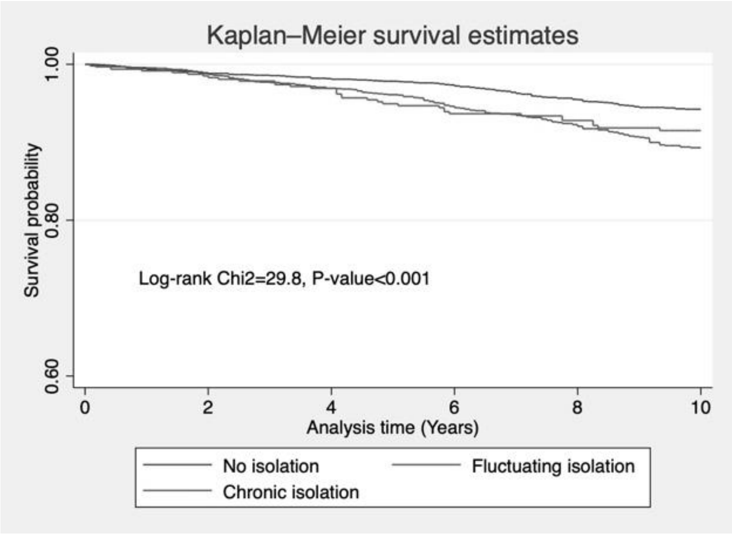

e

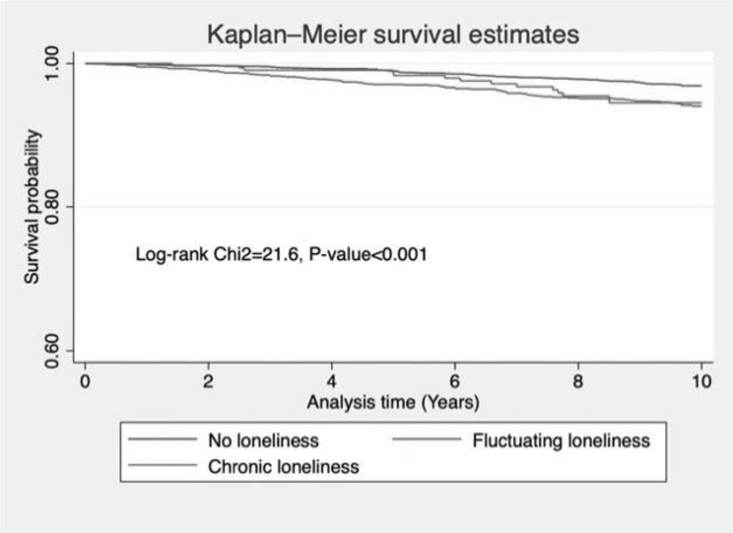

f

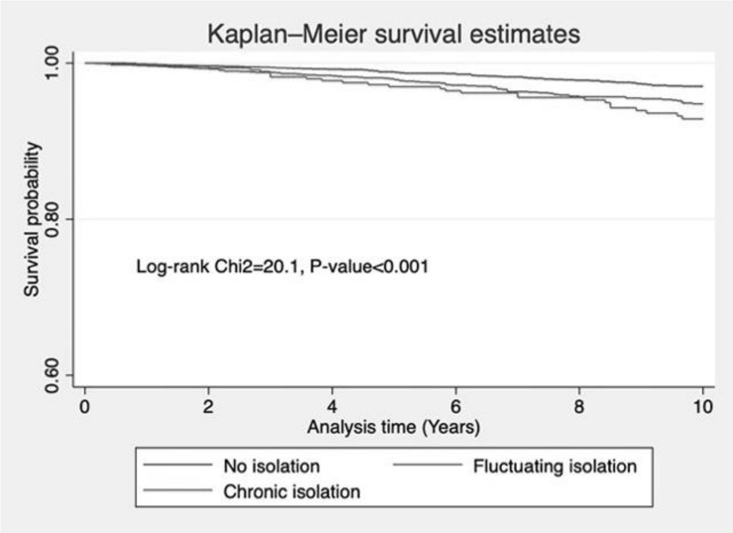

† **a.** Kaplan-Meier survival curve for 10-year cancer mortality by different patterns of loneliness. **b.** Kaplan-Meier survival curve for 10-year cancer mortality by different patterns of social isolation. **c.** Kaplan-Meier survival curve for 10-year cardiovascular disease (CVD) mortality by different patterns of loneliness. **d.** Kaplan-Meier survival curve for 10-year CVD mortality by different patterns of social isolation. **e.** Kaplan-Meier survival curve for 10-year respiratory disease mortality by different patterns of loneliness. **f.** Kaplan-Meier survival curve for 10-year respiratory disease mortality by different patterns of social isolation. Kaplan-Meier survival curves were graphed, and two-sided Log-rank tests were conducted to compare the differences in curves across patterns of loneliness/isolation.

Supplementary Figure 2 | Study sample selection process

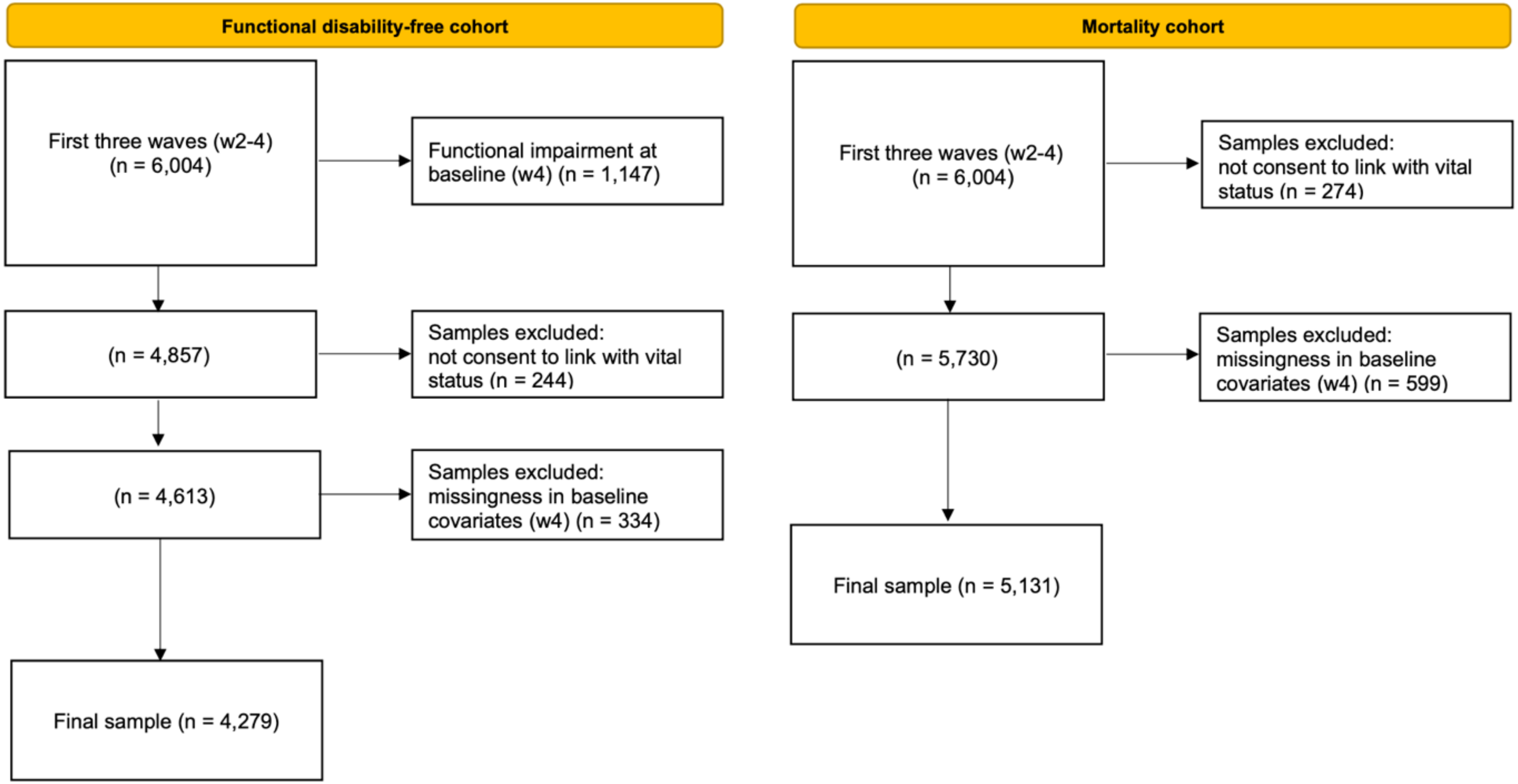

Supplement: Supplementary file 1 — Supplementary Figs. 1 and 2. [file 44220_2025_436_MOESM1_ESM.pdf]
