# Supplementary material for: Chronic loneliness and isolation phenotypes, incident functional impairment and mortality in England between 2004 and 2018
Source: Nat Ment Health. 2025 May 19;3(6):667–74. doi: 10.1038/s44220-025-00436-0 (PMC12165843; doi:10.1038/s44220-025-00436-0)
Supplement: Supplementary file 2 — Reporting Summary [file 44220_2025_436_MOESM2_ESM.pdf]

nature portfolio

Corresponding author(s): Qian Gao

Last updated by author(s): Apr 11, 2025

Reporting Summary

Nature Portfolio wishes to improve the reproducibility of the work that we publish. This form provides structure for consistency and transparency in reporting. For further information on Nature Portfolio policies, see our Editorial Policies and the Editorial Policy Checklist.

Statistics

For all statistical analyses, confirm that the following items are present in the figure legend, table legend, main text, or Methods section.

n/a	Confirmed	
☐	☒	The exact sample size (*n*) for each experimental group/condition, given as a discrete number and unit of measurement
☐	☒	A statement on whether measurements were taken from distinct samples or whether the same sample was measured repeatedly
☐	☒	The statistical test(s) used AND whether they are one- or two-sided<br>*Only common tests should be described solely by name; describe more complex techniques in the Methods section.*
☐	☒	A description of all covariates tested
☐	☒	A description of any assumptions or corrections, such as tests of normality and adjustment for multiple comparisons
☐	☒	A full description of the statistical parameters including central tendency (e.g. means) or other basic estimates (e.g. regression coefficient) AND variation (e.g. standard deviation) or associated estimates of uncertainty (e.g. confidence intervals)
☐	☒	For null hypothesis testing, the test statistic (e.g. *F*, *t*, *r*) with confidence intervals, effect sizes, degrees of freedom and *P* value noted<br>*Give P values as exact values whenever suitable.*
☒	☐	For Bayesian analysis, information on the choice of priors and Markov chain Monte Carlo settings
☒	☐	For hierarchical and complex designs, identification of the appropriate level for tests and full reporting of outcomes
☐	☒	Estimates of effect sizes (e.g. Cohen's *d*, Pearson's *r*), indicating how they were calculated

Our web collection on statistics for biologists contains articles on many of the points above.

Software and code

Policy information about availability of computer code

Data collection	No software was used for data collection.
Data analysis	All data analyses used open-source STATA code (i.e. Cox proportional hazards model and Fine-Gray competing risk model). All analyses were performed using STATA 17.0.

For manuscripts utilizing custom algorithms or software that are central to the research but not yet described in published literature, software must be made available to editors and reviewers. We strongly encourage code deposition in a community repository (e.g. GitHub). See the Nature Portfolio guidelines for submitting code & software for further information.

Data

Policy information about availability of data

All manuscripts must include a data availability statement. This statement should provide the following information, where applicable:

- Accession codes, unique identifiers, or web links for publicly available datasets
- A description of any restrictions on data availability
- For clinical datasets or third party data, please ensure that the statement adheres to our policy

ELSA data was available through registration with the UK data service (https://beta.ukdataservice.ac.uk/datacatalogue/series/series?id=200011).

Research involving human participants, their data, or biological material

Policy information about studies with human participants or human data. See also policy information about sex, gender (identity/presentation), and sexual orientation and race, ethnicity and racism.

Reporting on sex and gender	The ELSA datasets had collected sex information. We reported the distribution of sex in Table 1. The fully adjusted Cox proportional hazards model and Fine-Gray competing risk model have adjusted for sex and other confounders (Tables 2-3).
Reporting on race, ethnicity, or other socially relevant groupings	The study reported the distribution of participants' educational levels, household income, and employment status in Table 1 and models adjusted for these factors (Tables 2-3).
Population characteristics	The study consisted of 4279 participants in the functional disability-free cohort (67.0 years, SD=9.6) and 5131 participants in the mortality cohort (mean age=67.6 years, SD=9.8). The disability-free samples had a 20.2% all-cause mortality rate, with 9.83 years of median follow-up (IQR 7.17 to 10.17). The all-cause mortality rate was 22.4% in the mortality cohort, with a median follow-up length of 9.83 years (IQR 6.67 to 10.08). In the mortality cohort, the prevalence of chronic loneliness and isolation were 6.6% and 9.3%, respectively. 34.4% of the participants had fluctuating loneliness, and 38.4% with fluctuating isolation. Chronic loneliness and isolation were slightly less prevalent in the disability-free cohort, with 5.5% and 8.4% of the sample reporting the issues. The characteristics of study samples are reported in Table 1.
Recruitment	The English Longitudinal Study of Ageing (ELSA) was designed to recruit a representative sample of adults aged 50 years and older living in private households in England. The ELSA survey was started in 2002 and then followed up every two years. This study is a secondary data analysis of ELSA, and the detailed information about data collection has been described elsewhere (see data availability source in the manuscript).
Ethics oversight	ELSA Wave 9 received ethical approval from the South Central-Berkshire Research Ethics Committee on May 10, 2018 (17/SC/0588). ELSA Wave 8 received ethical approval from the South Central-Berkshire Research Ethics Committee on September 23, 2015 (15/SC/0526). ELSA Wave 7 received ethical approval from the National Research Ethics Service Committee South Central-Berkshire on November 28, 2013 (13/SC/0532). ELSA Wave 6 received ethical approval from the National Research Ethics Service Committee South Central-Berkshire on November 28, 2012 (11/SC/0374). ELSA Wave 5 received ethical approval from the Berkshire Research Ethics Committee on December 21, 2009 (09/H0505/124). ELSA Wave 4 received ethical approval from the National Hospital for Neurology and Neurosurgery and Institute of Neurology Joint Research Ethics Committee on October 12, 2007 (07/H0716/48). ELSA Wave 3 received ethical approval from the London Multi-Centre Research Ethics Committee on October 27, 2005 (05/MRE02/63). ELSA Wave 2 received ethical approval from the London Multi-Centre Research Ethics Committee on August 12, 2004 (MREC/04/2/006). Informed consent was acquired from all participants.

Note that full information on the approval of the study protocol must also be provided in the manuscript.

Field-specific reporting

Please select the one below that is the best fit for your research. If you are not sure, read the appropriate sections before making your selection.

☐ Life sciences ☒ Behavioural & social sciences ☐ Ecological, evolutionary & environmental sciences

For a reference copy of the document with all sections, see nature.com/documents/nr-reporting-summary-flat.pdf

Behavioural & social sciences study design

All studies must disclose on these points even when the disclosure is negative.

Study description	This quantitative study examined the roles of chronic loneliness and social isolation in predicting the onset of physical functional declines and all-cause and cause-specific mortality over 10-year follow-ups using a nationally representative sample of older adults living in England.
Research sample	The study used a nationally representative sample of older adults living in England, including study participants from different sociodemographic characteristics and health conditions. The English Longitudinal Study of Ageing (ELSA) survey was started in 2002 and then followed up every two years. ELSA is a national cohort study of more than 11,000 older participants aged 50+ living in England. In this study, we used data from wave 2 (in 2004), as this is the first wave that provides loneliness and social isolation measures. ELSA waves 2-9 were analysed. We linked the records with the UK Office for National Statistics (ONS) data to ascertain death dates and vital status and excluded loss to follow-up samples. Finally, the study consisted of 4279 participants in the functional disability-free cohort and 5131 participants in the mortality cohort. The proportion of gender and age distribution, etc., is reported in Table 1. Details of the study sample selection process are shown in Supplementary Figure 2.
Sampling strategy	The English Longitudinal Study of Ageing (ELSA) was designed to recruit a representative sample of adults aged 50 years and older living in private households in England. Further information on the sampling strategy can be found in the data resource. To enable analysis of mortality, we linked the records with the UK ONS, in which we excluded those who did not consent to linkage with the ONS data. The sample is sufficient as ELSA is a national study, and the age group reflects the ageing population.

Data collection	This study is a secondary data analysis of the ELSA, and no data collection was involved in this study. Data collection procedures can be found in the data resource.
Timing	This longitudinal study used panel data from the English Longitudinal Study of Ageing (ELSA), including 14 years of follow-up (waves 2-9, 2004-2018): Wave 2 (2004/05), Wave 3 (2006/2007), wave 4 (2008/09), Wave 5 (2010/11), Wave 6 (2012/13), Wave 7 (2014/15), Wave 8 (2016/17), and Wave 9 (2018/19).
Data exclusions	The study excluded data where participants had not responded to all measures (Functional disability-free cohort n=334; Mortality cohort n=599). The missingness was largely due to loss to follow-up and non-response. To enable data analysis of mortality, we also excluded those who did not consent to linkage with the UK Office for National Statistics (ONS) data (Functional disability-free cohort n=244; mortality cohort n=274). The exclusion criteria were pre-established.
Non-participation	To explore the relationship with functional decline, we limited the sample to participants free of physical disabilities at baseline (wave 4; see 'measures' below for definition) (n=4857). To enable analysis of competing risks of disability with death, we then excluded those who did not consent to linkage with the UK Office for National Statistics (ONS) data (n=244) and with missingness in covariates (n=334), leading to a final sample (n=4279). To explore the relationship with mortality, we linked the records with the UK ONS to ascertain death dates and vital status and excluded loss to follow-up samples (n=274). The study also excluded those with missingness in covariates (n=599), providing a final analytical sample of 5131. Details of the study sample selection process are shown in Supplementary Figure 2.
Randomization	This longitudinal study adjusted for age, sex, education, household income, employment, sedentary lifestyle, depression, persistent pain, and long-term illness in modelling.

Reporting for specific materials, systems and methods

We require information from authors about some types of materials, experimental systems and methods used in many studies. Here, indicate whether each material, system or method listed is relevant to your study. If you are not sure if a list item applies to your research, read the appropriate section before selecting a response.

Materials & experimental systems

n/a	Involved in the study	
☒	☐	Antibodies
☒	☐	Eukaryotic cell lines
☒	☐	Palaeontology and archaeology
☒	☐	Animals and other organisms
☒	☐	Clinical data
☒	☐	Dual use research of concern
☒	☐	Plants

Methods

n/a	Involved in the study	
☒	☐	ChIP-seq
☒	☐	Flow cytometry
☒	☐	MRI-based neuroimaging

Plants

Seed stocks	*Report on the source of all seed stocks or other plant material used. If applicable, state the seed stock centre and catalogue number. If plant specimens were collected from the field, describe the collection location, date and sampling procedures.*
Novel plant genotypes	*Describe the methods by which all novel plant genotypes were produced. This includes those generated by transgenic approaches, gene editing, chemical/radiation-based mutagenesis and hybridization. For transgenic lines, describe the transformation method, the number of independent lines analyzed and the generation upon which experiments were performed. For gene-edited lines, describe the editor used, the endogenous sequence targeted for editing, the targeting guide RNA sequence (if applicable) and how the editor was applied.*
Authentication	*Describe any authentication procedures for each seed stock used or novel genotype generated. Describe any experiments used to assess the effect of a mutation and, where applicable, how potential secondary effects (e.g. second site T-DNA insertions, mosiacism, off-target gene editing) were examined.*
